# A Curiosity of Immunisation

**Published:** 1905-03-25

**Authors:** 


### A CURIOSITY OF IMMUNISATION.

After the administration of a bacillary toxin the recipient will be, as regards his susceptibility to the particular body in question, in one of three conditions. His susceptibility may be as it was, or it may be heightened or lowered. The common lowering of susceptibility is of course what is desired, and in many cases obtained; it is what is called "active immunity." But occasionally there appears a heightened susceptibility, a condition to which C. Richet has given the name of "anaphylaxis," as representing the contrary of prophylaxis. Professor Lépine [1] has collected some facts touching this state of anaphylaxis, which, considering the importance which active immunisation against disease appears to be achieving in the hands of Professor Wright and others, deserve the best attention.

Metchnikoff has observed at the Pasteur Institute among horses which have been immunised against the diphtheria bacillus until they yield a good antitoxic serum, that occasionally such a horse on receipt of an ordinary injection of the poison will sicken and die. Knorr has recorded cases where rabbits injected daily with one-tenth of an ordinary lethal dose of tetanus toxin have died before they have in all received as much as one lethal dose. V. Behring and Kitashima have observed the same thing in horses under a course of immunisation against the diphtheria bacillus—viz, that if the poison be given at intervals in small doses the animal may die before the equivalent of one lethal dose, given at one time, has been attained. This appears an anomalous state of things, for at the time of its death the animal may be yielding a strongly immune serum. It seems that though the vital manufacture of protecting substances is enhanced, the susceptibility of the organism is disproportionately heightened.

Applying these facts to practice, Professor Lépine finds in them an explanation of the intense reaction which follows the injection of tuberculin in a tuberculous subject—and the new tuberculin is nothing but the pounded bodies of dead tubercle bacilli. Professor Wright has drawn attention to the temporary drop in specific resistance which follows upon an injection of tuberculin, and has dwelt upon the danger of repeating the dose at random, that is to say, without having first determined the effect of the initial one, for he has demonstrated that if a second injection be made during such a phase of anaphylaxis, or heightened susceptibility, the patient may be made much worse, or even die. There seems little doubt that part at all events of the discredit attached to the early days of tuberculin treatment depended on the lack of precise information as to the state of the patient's specific resistance at the times of injection. Professor Wright claims that this want is removed by a method whereby it is possible to estimate the state of specific resistance in any individual by counting the number of tubercle bacilli ingested by a given number of leucocytes from the patient's blood. A comparison at intervals of the figures thus obtained will reveal whether the resistance is rising or dropping, and will therefore indicate whether or no it is wise at the moment to repeat the inoculation.

Professor Lépine considers that in the case of influenza this phase of diminished resistance is particularly prone to appear; an opinion founded on his experience that the later attacks of this disease are generally more severe than the early ones. In the case of pneumonia, on the other hand, although it is well known that one attack often appears to predispose the victim to subsequent ones, there does not seem to be any evidence, in his opinion, that the type of the disease tends to advance in gravity with repetition.

[1] La Semaine Med., March 1, 1905. *See* also Jour. of R.A.M.C., March 1905, p. 313.

